# Individual Differences in Verb Bias Sensitivity in Children and Adults With Developmental Language Disorder

**DOI:** 10.3389/fnhum.2019.00402

**Published:** 2019-11-19

**Authors:** Jessica E. Hall, Amanda Owen Van Horne, Thomas A. Farmer

**Affiliations:** ^1^Speech, Language, and Hearing Sciences, The University of Arizona, Tucson, AZ, United States; ^2^Communication Sciences and Disorders, University of Delaware, Newark, DE, United States; ^3^Department of Psychology, California State University, Fullerton, Fullerton, CA, United States

**Keywords:** developmental language disorder, sentence processing, statistical learning, language development, mouse tracking, verb bias, artificial grammar learning, specific language impairment

## Abstract

A number of experiments support the hypothetical utility of statistical information for language learning and processing among both children and adults. However, tasks in these studies are often very general, and only a few include populations with developmental language disorder (DLD). We wanted to determine whether a stronger relationship might be shown when the measure of statistical learning is chosen for its relevance to the language task when including a substantial number of participants with DLD. The language ability we measured was sensitivity to verb bias – the likelihood of a verb to appear with a certain argument or interpretation. A previous study showed adults with DLD were less sensitive to verb bias than their typical peers. Verb bias sensitivity had not yet been tested in children with DLD. In Study 1, 49 children, ages 7–9 years, 17 of whom were classified as having DLD, completed a task designed to measure sensitivity to verb bias through implicit and explicit measures. We found children with and without DLD showed sensitivity to verb bias in implicit but not explicit measures, with no differences between groups. In Study 2, we used a multiverse approach to investigate whether individual differences in statistical learning predicted verb bias sensitivity in these participants as well as in a dataset of adult participants. Our analysis revealed no evidence of a relationship between statistical learning and verb bias sensitivity in children, which was not unexpected given we found no group differences in Study 1. Statistical learning predicted sensitivity to verb bias as measured through explicit measures in adults, though results were not robust. These findings suggest that verb bias may still be relatively unstable in school age children, and thus may not play the same role in sentence processing in children as in adults. It would also seem that individuals with DLD may not be using the same mechanisms during processing as their typically developing (TD) peers in adulthood. Thus, statistical information may differ in relevance for language processing in individuals with and without DLD.

## Introduction

Statistical learning is often studied in the context of language learning because researchers have considered statistical learning tasks as representative of the types of tasks that people face when learning language. Language is full of statistical regularities of different types, and statistical learning tasks can isolate some of these features to determine how they are learned at different points in development and in different populations. Indeed, individual differences studies have documented that variability in statistical learning ability can predict variability in performance on measures of language comprehension. Misyak and Christiansen, 2012 and Misyak et al. (2010) studies with adults, and Kidd (2012) and Kidd and Arciuli (2016) studies with children, for example, have shown positive correlations for performance on statistical learning tasks and comprehension tasks. Additionally, Lany et al. (2018) found evidence of a relationship between segmenting ability in a statistical learning task and how efficiently infants processed speech.

The relationship between statistical learning and language ability has been important for understanding developmental language disorder (DLD). DLD, formerly known as Specific Language Impairment, is a disorder that affects an individual’s ability to effectively learn and use language. This deficit in language learning and use is not attributable to any other biomedical cause. It affects approximately 13% of children (when including children with low non-verbal intelligence scores as the new terminology of DLD mandates; Tomblin et al., 1997; Bishop et al., 2017) and has lifelong academic and social consequences (Conti-Ramsden et al., 2018). Several researchers have posited that learning the statistics of language may be a barrier for people with DLD and may have some causal explanation for the profiles we see in DLD. One of the first studies of statistical learning in DLD showed a relationship between statistical learning and receptive vocabulary (Evans et al., 2009). Arciuli and Conway (2018), however, recently argued that “… research on statistical learning and language acquisition in developmental disabilities should be broadened beyond group comparisons to consideration of individual differences and contextual factors that contribute to variability in language difficulties within and across disabilities” (p. 7). We agree that it is important to consider and test the relationship between statistical learning and language ability, especially given that recent research is casting some doubt onto what statistical learning tasks can reveal about language. For example, Spit and Rispens (2019) found that although gifted children showed better comprehension of object relative clauses than their same-age peers, performance on a serial reaction time task did not account for this variability. This finding is evidence that statistical learning *in general* may not have a strong relationship with language ability. The modality of the statistical learning task, especially if it differs from the language task, could potentially mask any relationship. Siegelman et al. (2018a) make a convincing argument that the vast experience people have with sounds in their native language impacts their performance on auditory learning tasks, unlike in visual statistical learning tasks in which people do not have similar amounts of entrenched experience. It is also possible that statistical learning involves a number of underlying components that enable encoding and abstraction, and that statistical learning tasks vary in how they test these components (Arciuli, 2017).

Three challenges contribute to difficulty in establishing reliable relationships between statistical learning ability and variability in language learning and processing. One is the problem of how to appropriately measure language proficiency, given that it entails numerous skills. Standardized tests of grammatical proficiency may cover too broad a range of constructs to demonstrate a strong relationship, with a limited number of items on any one skill or knowledge type. Given that the multi-factorial nature of global proficiency tasks may reduce the likelihood of detecting relationships to statistical learning ability, a more appropriate strategy for empirically documenting such links involves employing language tasks designed to measure more narrow (sub)components of grammatical competency. For example, Misyak et al. (2010) documented such a relationship by utilizing a non-adjacent dependency learning task to predict the ease of processing relative clauses, a language task that involves tracking the non-adjacent dependency between the embedded and main verbs of the sentence.

Secondly, there is the appropriateness of the statistical learning task. For example, the serial reaction time task has been used many times, but we question its relevance to language learning for reasons of modality and the statistics involved. Kidd et al. (2018) make this point clearly in their paper: “Typically, studies quantify SL [statistical learning] as the ability to learn simple transitional probabilities, but SL-for-language likely requires more than this…” (Box 1, p. 163). Erickson and Thiessen (2015) also note that “language acquisition involves sensitivity to more kinds of statistical information than simple transitional probabilities” (p. 68) in their discussion of underlying processes of extraction and integration. Proficient language use does not end with learning that *the* always predicts *boy*, but requires learning that *the* predicts a set of words that share a syntactic distribution and semantic features. Statistical learning tasks should be chosen based on the relevance of the potential component mechanisms for the language skill being studied (as per the non-adjacent dependency example discussed in the preceding paragraph).

Relatedly, we take the view that language learning involves learning multiple types of probabilistic relationships among units existing at multiple representational levels. Thus accordingly, because language develops over time, we might expect statistical learning to correlate differently to language ability at different developmental time points (i.e., relationships may appear stronger after a skill is mastered than before). To this point, Arnon (2019) found reliability across three statistical learning tasks in adults but not in children. Thus, the final challenge is to choose the appropriate task for the age group being tested, taking into consideration theories of language acquisition and cognitive development in tandem.

We addressed these three challenges through the following study design. First, we designed tasks that seemed to rely upon one common skill: the ability to learn a word’s syntactic distribution. For our language task, we used an adaptation of Snedeker and Trueswell’s (2004) task that captures verb bias sensitivity. Verb bias is the product of a certain type of grammatical category learning, the learning that some verbs are more likely to occur with specific words, phrases, or interpretations. For the statistical learning task, we chose an auditory statistical learning task that employed linguistic stimuli. We used an adaptation of the artificial grammar learning experiment from Reeder et al. (2013) that focuses on grammatical category learning. We used this design in experiments with children (Hall et al., 2018a) and child and adult populations with DLD (Hall et al., 2017, 2018b). Because distributional learning drives successful performance on the artificial grammar learning task and because of the distributional nature of the verb bias information for differentiated performance, we think these tasks tap into similar underlying components of grammatical processing and representational knowledge. Accordingly, we predict a significant relationship between scores on these two measures. We employed an implicit measure of grammatical comprehension ability, mouse tracking, to capture variability in sentence processing at a more fine-grained level than provided by off-line indices of comprehension. Preliminarily, we have examined use of verb bias by college students with DLD (Hall et al., 2019) and found that they were less sensitive than typically developing (TD) peers, suggesting indeed that the presence of a language disorder may be related in some way to deficits in the processing and representation of verb bias.

Finally, we chose an age group in which we expected these skills to be nearing adult proficiency but possibly impaired for children with DLD: ages 7–9. Snedeker and Trueswell (2004) showed that children as young as five were sensitive to verb bias, but verb bias had not yet been studied in children with DLD. Peter et al. (2015) showed evidence of stronger verb bias effects with age in a study of adults and children ages 3–6. At 7–9, children are just beginning to read, and reading may be a factor that further entrenches verb bias (see Perfetti et al., 2001; Shankweiler et al., 2008; Mani and Huettig, 2014; for evidence of a relationship between literacy and sentence processing). We hoped to minimize the impact of reading and so we did not choose older children. We did not choose younger ages because, as Peter et al. (2015) state, early on, verb bias is much weaker in young children because their cumulative experience is smaller, and thus more susceptible to “random fluctuations in the input.” Fluctuations could take the form of uncommon constructions in children’s storybooks and songs or in the speech of peers. These studies, in combination with findings from studies that show the manipulability of verb bias (Wells et al., 2009; Farmer et al., 2011; Fine and Jaeger, 2013; Fine et al., 2013; Ryskin et al., 2017), suggest that verb biases are still forming in children as old as 9 and continue to be shaped by experience throughout the lifespan. Further evidence of prolonged syntactic development throughout adolescence comes from brain imaging studies of sentence processing (Schneider et al., 2016, 2018; Schneider and Maguire, 2018).

Although we are interested in group differences, we took seriously the call of Kidd et al. (2018) to test this relationship with a more rigorous methodological approach. We elected to use a multiverse approach to more transparently characterize our findings. The multiverse is a relatively new method for analyzing and reporting data to increase transparency and rigor (Steegen et al., 2016). In the multiverse approach, the many choices that the researcher must make about data processing and selection are made plain. For example, options such as removing outliers or not, classifying SES by income alone or by income plus years of education, and binning participants into subgroups according to a numerical measure, among many others, are all presented, and the data are then analyzed for each option. By presenting all possible results (the multiverse), the researcher communicates a more accurate assessment of the robustness of the findings. Note that the multiverse approach is not a method for selecting or evaluating models; instead, it is a principled way to show the strength of findings given the number of arbitrary choices researchers must make when analyzing complex datasets. The multiverse approach is a method of avoiding both Type 1 and Type 2 errors because it allows a wider lens through which to view the data. We elected to use the multiverse given the large and confusing number of measures that could be used to determine learning within each task.

One challenge in using a multiverse approach is that the complexity involved nearly necessitates using data that are already available in the literature. With the exception of children’s performance on the verb bias task, all data in this paper have been previously reported. This includes sensitivity to verb bias by adults with and without DLD (Hall et al., 2019) and artificial grammar learning by both children with and without DLD (Hall et al., 2018a,b) and adults with and without DLD (Hall et al., 2017). Study 1 provides the findings for how children with and without DLD perform on the verb bias task, completing the data reporting required for the multiverse analysis. In Study 1, we draw on the previously reported adult data to make clear the range of performance and possible predictors of performance and to support interpretation of the child data. In Study 2 we then report our findings using the multiverse approach to determine evidence of a relationship between statistical learning and language.

## Study 1 Introduction

Snedeker and Trueswell (2004) used a visual world paradigm to determine differences in verb bias and visual referent sensitivity between TD adults and children. In their study, they used syntactically ambiguous sentences (e.g., *Feel the frog with the feather*) that required children to act out one of the two possible interpretations (either using the object as an *instrument* to complete the action or choosing an animal that was holding the object in which it is as seen as a *modifier*). Stimuli varied by the likelihood that the verb in the sentence was to appear with one of the two interpretations in corpus and sentence-completion norming data (*poke* occurs more often in instrument interpretations whereas *hug* occurs more often in modifier interpretations), or by the number of visual referents present (one frog vs. two frogs). TD children showed no differences in verb bias sensitivity compared with adults in both their choice of interpretation and in eye tracking measures of where they looked while completing the task. However, children did show different patterns of choice and looking behavior than adults when two referents were present.

We adapted this task for use with mouse tracking in our study of verb bias sensitivity in college students with DLD (Hall et al., 2019). In our study, participants viewed a computer screen with illustrations of each interpretation of the ambiguous sentence (e.g., *The elephant pokes the camel with the feather*) in the two top corners. Participants were instructed to click on the interpretation that went with the sentence. We used the trajectory of their mouse movement to measure the amount that they were attracted toward the competing picture for a given trial. Previous mouse tracking studies have demonstrated that when very little competition is present, participants move the mouse in a straight trajectory toward their choice; whereas trials with a great amount of competition result in trajectories that curve toward the competitor (Spivey et al., 2005; Farmer et al., 2007; Spivey, 2007; see Freeman et al., 2011, for an overview). Thus, mouse trajectories provide a means to continuously measure dynamic competition during sentence processing through time-normalized *x,y* coordinates. In this way, we could measure the role of verb bias in the choice of interpretation and in the process of making that choice. We found that TD college students chose interpretations that were consistent with verb bias more often than their peers with DLD, and their mouse trajectories also reflected greater sensitivity to verb bias, with more curved mouse trajectories when they chose an interpretation that was inconsistent with verb bias than when it was consistent. The trajectories for the group with DLD did not show this pattern.

Two previous studies have shown poorer verb comprehension in children with language deficits relative to TD peers. Kelly and Rice (1994) showed that children with DLD had no preference for a change-of-state vs. motion interpretations for a novel verb form, whereas age-matched TD peers demonstrated a preference for change-of-state interpretations, suggesting that the children with DLD may not be as sensitive to subtle aspects of verb subcategory that lead TD children to have a change-of-state bias. Nation et al. (2003) showed that children with poor comprehension skills were as quick to anticipate the object of a verb as TD peers for verbs with specific semantic restrictions (e.g., “eat” predicts something edible). However, these participants spent less time overall looking at the target object than more skilled comprehenders. This is further evidence that although children with DLD may be sensitive to restrictive semantics associated with verbs, they may not have well-developed preferences for interpretations of verbs based on statistical likelihood.

In this study, we examine the degree to which grammar production skills, as measured by the Structured Photographic Expressive Language Test, Third Edition (SPELT-3, Dawson et al., 2003), a test that can diagnostically discriminate between children with and without DLD, and text exposure, as measured by the recognition of children’s book titles, predict verb bias sensitivity in children ages 7–9. We predict that, like the adults with DLD in our previous study, children with lower SPELT-3 scores will show less sensitivity to verb bias than more grammatically productive peers, both in the choices they make and at the level of cognitive competition, as revealed by mouse-trajectories. We might expect more differences in childhood than in adulthood because adults could have developed compensatory strategies to aid them in sentence processing. We examine the role of text exposure because we expect that children who read or are read to more often will have more entrenched verb biases than children with less reading experience.

## Study 1 Methods

### Participants

Participants were 55 children ages 7–9, 19 who were classified as having DLD and 36 who were classified as TD. All of the participants with DLD and 22 of the TD participants in this study also participated in the statistical learning study reported in Hall et al. (2018b); their demographics are reported in the first two rows of Table 1 for the purpose of data transparency. Thirty-four of the 36 TD children participated in the study reported in Hall et al. (2018a). Six children with DLD participated in the current study after completing a treatment study on morpheme production (Owen Van Horne et al., 2017, 2018). All children had normal hearing verified by a screening (American Speech-Language-Hearing Association, 1997), had normal or corrected vision, passed a non-verbal intelligence test (Kaufman Brief Intelligence Test-2, matrices; Kaufman and Kaufman, 2004), and had no history of autism spectrum disorders or neurological disorders by parent report. Children in the DLD group scored a standard score of 95 or below on the SPELT-3 (Dawson et al., 2003). Although our children were older ages than those in Perona et al. (2005), which recommends the 95 cut off score for highest sensitivity and specificity, we think that this categorization is reasonable because all but three children in the DLD group received services for speech and language. Of those three, two received reading services. Children in the TD group scored above 95 on the SPELT-3 and had no history of speech or language difficulties. Children in both groups completed the Peabody Picture Vocabulary Test, 4th edition (PPVT-4, Dunn and Dunn, 2007), and the title recognition task (Montag and MacDonald, 2015). In the title recognition task, designed to measure the amount children ages 8–12 are exposed to text, the examiner reads aloud titles of real and fake children’s books, and the child responds yes if they have heard of that book before. Data were screened as described in section “Data Screening,” and data from three TD and three DLD participants were excluded. Participant demographics and test scores for those 49 participants included in the final analyses are reported in Table 1.

**TABLE 1:** Participant demographic and testing information by diagnostic category (DLD, developmental language disorder; and TD, typically developing), after excluding participants as described in the screening measures of section “Data Screening,” for child datasets in Studies 1 and 2.

					**Maternal**											
			**Age**		**education**											
			**(months)**		**(years)**		**PPVT-4 raw**		**PPVT-4 SS**		**SPELT-3 SS**		**KBIT-2 SS**		**TRT**	
									
	***n***	***n* male**	***M***	***SD***	***M***	***SD***	***M***	***SD***	***M***	***SD***	***M***	***SD***	***M***	***SD***	***M***	***SD***
DLD	16	12	92.9	8.6	15.0	2.2	133.1	13.0	103.8	9.2	84.3	10.1	106.8	14.3	–7.1	7.0
Age-matched TD	17	9	95.2	8.0	17.1	3.5	153.6	11.1	119.6	12.6	110.3	7.7	117.2	11.6	–2.2	6.9
Additional TD	16	4	105.1	14.9	18.2	4.4	162.9	19.0	120.9	11.1	111.4	5.7	109.4	13.1	4.0	12.5

*PPVT, Peabody Pictures Vocabulary Test, 4th Edition. Raw, raw scores; SS, standard scores; SPELT-3, Structured Photographic Expressive Language Test, 3rd Edition; K-BIT 2, Kaufman Brief Intelligence Test, 2nd Edition, non-verbal subtest. Scores on the title recognition task (TRT) are out of a range of −30 to 30, with −30 being the lowest score.*

### Materials

We again used the mouse tracking adaptation of the visual world paradigm task from Snedeker and Trueswell (2004) that we used in Hall et al. (2019). Sentences in the experimental trials were syntactically ambiguous, e.g., “The giraffe brushes the zebra with the sponge.” Two possible interpretations were displayed, an instrument interpretation (the giraffe using a sponge to brush the zebra) and a modifier interpretation (the giraffe using its foot to brush a zebra that holds a sponge). We compared mouse trajectory curvature on trials when participants chose an interpretation that matched the verb bias vs. trials in which the choice did not match bias. We defined sensitivity to verb bias as greater mouse curvature on mismatched trials than matched trials. We also had comprehension trials which displayed only one of the two correct interpretations. The alternative picture showed an impossible interpretation (the giraffe holding a sponge but not brushing the zebra). These trials provided a screener for children who did not understand the sentences. We expected more incorrect trials in the DLD group due to comprehension difficulties, though these should still be somewhat rare in both groups because of the simple nature of sentences. Pictures across all trial types were made to appear as similar as possible, with the object and animals roughly the same size so as to neutralize the salience of items in the visual display. Verbs in the sentences were either biased to appear with instrumental phrases (The butterfly hits the grasshopper with the flower) or biased to appear with modifier phrases (The gorilla hugs the cat with the blanket), based on the norming data and classification reported in Snedeker and Trueswell (2004). Filler trials had two pictures of different animals and sentences that asked participants to “Click on the (animal name) that (animal attribute).” These were used as a measure for overall mouse movement because DLD is associated with poorer motor control (Hill, 2001) and were entered in our models as a covariate. Practice trials in the beginning required matching color and shape (“The red circle is bigger than the blue”) and familiarized participants with the task. See the Appendix in Hall et al. (2019) for a description of experimental sentence and picture stimuli.

Participants completed eight practice trials first, and then 16 experimental trials, 16 comprehension trials, and 24 fillers for a total of 56 items, presented in a completely randomized order. Pictures were counterbalanced for position of the modifier and instrument interpretation, position of the impossible interpretation on comprehension trials, and position of the correct interpretation on filler trials. Within each of the experimental and comprehension trials there were eight sentences with instrument-biased verbs and eight with modifier-biased verbs. Direct objects in the sentences were from Snedeker and Trueswell (2004) and were chosen to have little impact on the interpretation of the sentence as instrument or modifier with the verb they appeared with.

We used MouseTracker software (Freeman and Ambady, 2010) to deliver the task and to measure mouse curvature on each trial. The mouse was reset to the same location at the beginning of the trial, and *x,y* coordinates were used to determine curvature. MouseTracker software calculates the maximum deviation of the mouse from an imaginary straight line drawn between actual start and end points of each mouse movement. Warning messages appeared at the end of a trial if the participant took longer than three seconds to initiate movement. The examiner read the message to the child and explained that she could look at the pictures as long as she liked *before* pressing the button, but she needed to choose as quickly as possible after she heard the sentence.

### Measures

Using the lme4 package (Bates et al., 2015) and the lmerTest package (Kuznetsova et al., 2017) in R version 3.5.1 (R Core Team, 2018), we ran linear mixed effects models to explore subject and linguistic factors influencing choice of interpretation and mouse trajectories for experimental trials.

#### Choice of Interpretation

Because the interpretations shown during experimental trials were both possible, we examined overt choice to determine children’s sensitivity to the bias of the individual verb. We were also curious if children would be sensitive to the global bias in which instrument interpretations are overall more likely in English. We used a mixed effects logistic regression with likelihood of instrument choice as the dependent variable and the bias of the verb as a fixed factor. SPELT-3 served as a measure of participants’ grammar production skills and the title recognition task as a measure of text exposure. Categorical variables were effects coded, and the reference variable was instrument verb. Continuous variables were centered. The maximal random effects structure included a subject slope for verb bias and intercepts for subject and item. Akaike information criterion (AIC) was used to determine model fit, using the maximal random effects structure when the difference between models’ AIC was less than 2.

Consistent with the results of five-year-old typical children in Snedeker and Trueswell (2004), we predicted that children would show sensitivity to the bias of individual verbs and a slight tendency to choose instrument interpretations more often, and reflecting knowledge of the global instrument bias in English. We predicted that this sensitivity would vary based on language proficiency and/or text exposure.

#### Mouse Movements

We used a linear mixed effects model in which the dependent variable was mouse curvature as measured by maximum deviation, and fixed effects included the bias of the verb, the consistency of choice of interpretation with verb bias, the expected strength of verb bias according to Snedeker and Trueswell (2004) norms (see Table 2), SPELT-3 scores, title recognition task scores, and interactions between consistency, bias, and proficiency measures. Strength of verb bias was a continuous variable used to account for variability in items attributed to the linguistic cue rather than the visual cues. We included participants’ average maximum deviation for filler trials to control for individual differences in motor control. For all analyses, variables were dummy coded, and instrument bias and consistent choice served as the reference categories. The maximal random effects structure included subject slopes for verb bias, consistency of choice of interpretation, their interaction, as well as strength of bias, and random subject and item intercepts. AIC was used to determine model fit using the same criterion as above. We included random effects and measures of motor control and strength of bias because the latter two are attributable and meaningful in the consideration of differences between individuals with and without DLD, and therefore not random.

**TABLE 2:** List of verbs by bias type and the nouns that appear with the verbs in the sentences.

**Bias**	**Verb**	**Instrument/modifier**	**Strength**
Instrument	Hit	Flower	24
	Tickle	Fan	24
	Poke	Feather	29
	Clean^∗^	T-Shirt	38
	Bop^∗^	Ball	38
	Brush^∗^	Sponge	43
	Cover^∗^	Book	43
	Feed^∗^	Glass	43
Modifier	Look at	Glass	19
	Hug	Blanket	19
	Find	Stick	24
	Talk to	Tube	24
	Sing to	Funnel	29
	Yell at^∗^	Funnel	30
	Listen to^∗^	Tube	38
	Choose^∗^	Fork	81

*Strength is determined by the percentage of time that the verb appeared with the biased interpretation in the norming sentence completion study reported in Appendix B of Snedeker and Trueswell (2004). Asterisks denote “strongly biased” verbs.*

We predicted that measures of proficiency would predict sensitivity to verb bias, with straighter trajectories (small maximum deviation) across all trial types representing poor use of verb bias information. Greater verb bias sensitivity would be demonstrated by straighter trajectories on trials in which interpretation choice was consistent with verb bias, and more curved trajectories (large maximum deviation) on trials when participants chose an interpretation inconsistent with bias.

### Procedures

This is the same experiment as was reported in Hall et al. (2019) and the same procedures were followed for children as the adults in that study. We briefly discuss them here. Procedures were approved by the Institutional Review Board at the University of Iowa. Children participated in this study during a one-hour session that sometimes also included other tasks, including standardized testing and the artificial grammar learning task reported in Hall et al. (2018a,b).

Children sat at a laptop computer and listened to the examiner give instructions. The examiner told children to carefully view the two pictures located at the top corners of the screen on each trial to find the differences between the pictures. When they saw the differences, they could then click on the Start button to begin the trial and listen to the sentence. Children were instructed to move the mouse as quickly as possible to the picture that went with the sentence after the sentence played. If children had difficulty with this, they were told to wait until the star appeared on the screen to move the mouse. Children were reminded to choose as quickly as possible several times during the experiment, and they were given stickers and encouragement and occasionally short breaks if their attention waned. All children completed the experiment with their right hand. Many children reported never having used a computer mouse before, so although we did not collect handedness information, it likely did not matter because children were not especially dexterous and because we included a measure of movement on control trials as a covariate.

## Study 1 Results

### Data Screening

We did not include practice trials in any analysis. We measured accuracy on comprehension trials to screen trials and participants. We excluded three participants with DLD and three TD participants for choosing incorrect interpretations for more than half of the 16 comprehension trials, leaving us with 37 participants. With these children excluded, a Mann-Whitney *U* test confirmed that participants with DLD had more incorrect responses than TD children, *U* = 113, *p* < 0.05. The average number of incorrect responses for a participant with DLD was 6.5 (*SD* = 1.3) and for TD children was 4.3 (*SD* = 2.3). Table 1 provides demographic information for non-excluded participants only.

We also screened each experimental trial mouse trajectory for the remaining participants for aberrant mouse movements (i.e., non-interpretable looping cycling leftward and rightward; Freeman et al., 2008; excluding 37 trials by children with DLD and 33 trials by TD children; 70 trials total). We also excluded trials with a reaction time exceeding 5000 ms (10 trials by children with DLD; 14 trials total); and trials in which initiation time exceeded 2000 ms (1 trial by children with DLD; 2 trials total). Overall, 18.8% of experimental trials for children with DLD and 7.4% of the TD children’s experimental trials, or 11.2% of total data, were discarded. We did not exclude any participants for missing trials because mixed effects models can adequately handle missing data. The children with the largest number of missing trials in each diagnostic group were a child with DLD with 7 missing trials and a TD child with 6 missing trials. The mean total number of missing trials for children with DLD was 3.0 (*SD* = 1.8) and 1.2 (*SD* = 1.3) for TD children, a difference that was significant according to a two-tailed independent sample *t*-test, *t*(47) = 4.00, *p* < 0.01.

Finally, we ran a linear mixed effects model with a random subject intercept with only filler trials included to test for baseline motor control differences between groups. There was no difference between children with and without DLD for maximum deviation of mouse trajectories on control trials, *p* = 0.48.

### Choice of Interpretation

**FIGURE 1:**
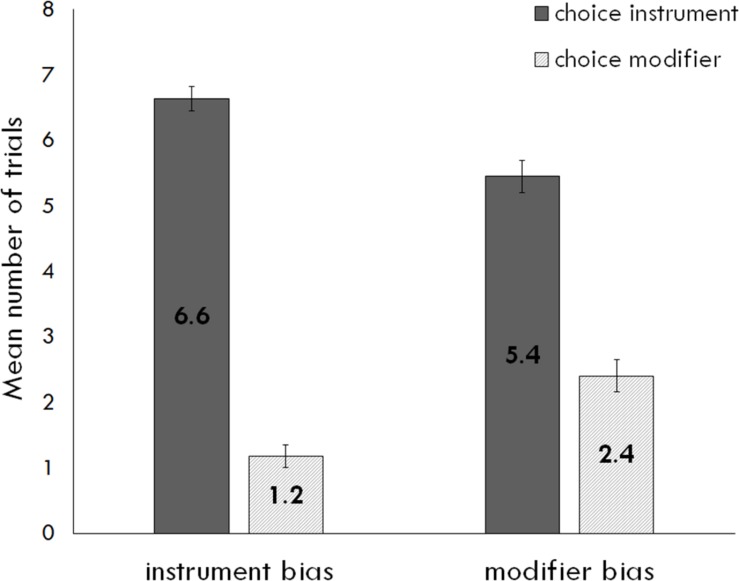
Mean number of trials (out of 8) in which participants chose each interpretation (instrument and modifier) for instrument- and modifier-biased trials. Error bars represent standard error.

We first examined choice of interpretation. The best fit model included random intercepts for subject and item. The dependent variable was the probability that a participant would select an instrument interpretation on a given trial. See Table 3 for log odds (reported as β) and Figure 1 for an illustration of means by trial type. Recall that the instrument interpretation is the more likely overall interpretation of “with the X” phrases in English. Children were sensitive to this global bias: participants were 82% likely to choose instrument on a given trial, *z* = 4.71, *p* < 0.0001. The bias of the verb did not significantly influence choice of interpretation, *z* = 1.65, *p* = 0.10, and measures of text exposure and language proficiency did not significantly interact with bias or have any effect on participants’ likelihood of choosing instrument, *p*s > 0.3.

**TABLE 3:** Summary of logistic regression analysis for variables predicting the probability that child participants choose instrument interpretation in the verb bias task.

**Factor**	**Variance**	***SD***	**β**	**SE**	***p***
**Random factors**					
Subject	0.65	0.81			
Item	0.89	0.94			
**Fixed factors**					
(Intercept)			1.50	0.32	<0.0001
Bias (reference category = instrument)			0.45	0.27	0.10
SPELT-3 standard score, centered			–0.01	0.01	0.60
Title recognition task, centered			–0.01	0.02	0.43
Bias × SPELT-3			0.01	0.01	0.33
Bias × Title recognition task			–0.01	0.01	0.54
Model χ^2^ = 5.92					

*SPELT-3, Structured Photographic Expressive Language Test, 3rd Edition.*

### Mouse Movements

Although we did not find that children’s grammatical proficiency or exposure to text influenced their choice of interpretation, it is possible they will predict sensitivity as represented in mouse trajectories. Sensitivity to bias in mouse trajectories would be indicated by a significant main effect or interaction with consistency of interpretation. This would be interpreted as more curved trajectories, and thus more competition, when participants chose responses that were inconsistent with bias.

**FIGURE 2:**
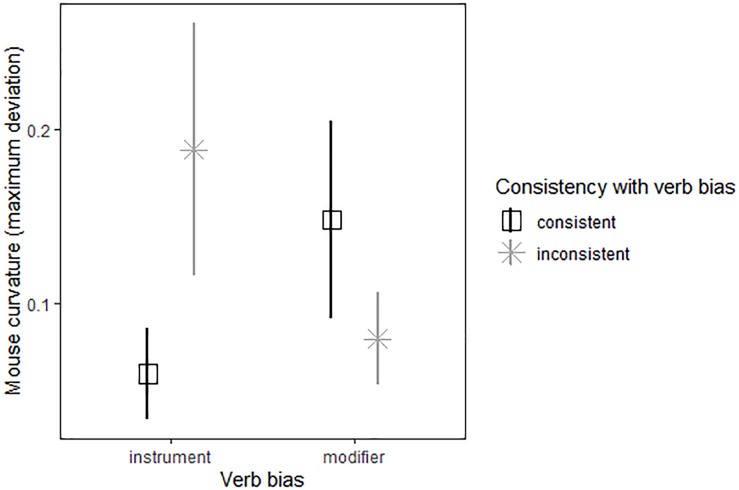
Mean maximum deviations of mouse trajectories by verb bias (instrument and modifier) and consistency of choice (consistent and inconsistent). Error bars represent standard error.

**FIGURE 3:**
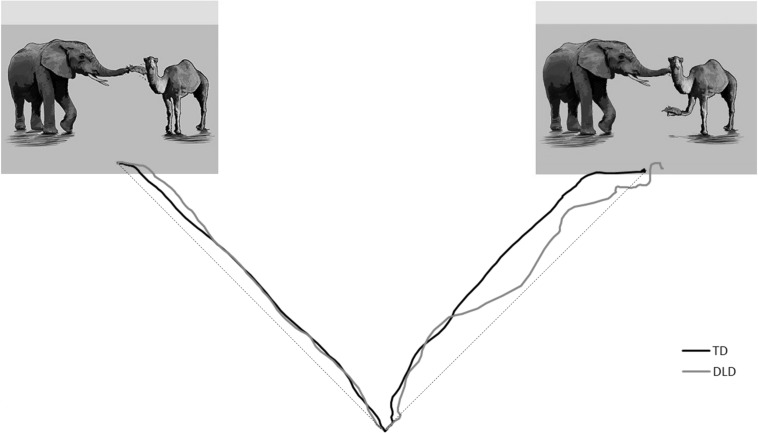
Average mouse trajectories for choosing instrument **(left)** vs. choosing modifier **(right)** interpretation on instrument-biased trials, with the group with developmental language disorder (DLD) shown in gray, and the group with typical development (TD) shown in black. The dashed lines represent the ideal straight line between trajectory start and endpoints from which maximum deviation for each trajectory is measured.

Model results are reported in Table 4 and mean maximum deviation of mouse trajectory by trial type illustrated in Figure 2. The best fit model included random subject slopes for bias and consistency. There were two significant factors: an interaction between bias and consistency, *t*(620.4) = 2.52, *p* = 0.01, and maximum deviation on control trials, *t*(87.8) = 2.75, *p* < 0.01. There was a marginal three-way interaction between bias, consistency, and the title recognition task which measured text exposure, *t*(663.1) = 1.70, *p* = 0.09, an effect which moved to *p* = 0.47 when the participant with the highest title recognition task score was removed from the dataset. Interpreting these effects, we found that participants showed a larger difference between choosing inconsistently vs. consistently on instrument-biased trials than on modifier-biased trials. Participants showed curved trajectories when choosing against bias on instrument-biased trials, but somewhat straight trajectories when choosing with bias. Figure 3 provides an illustration of averaged mouse trajectories by diagnostic group on trials with instrument-biased verbs, with choosing consistently with bias shown on the left and choosing against bias shown on the right. Participants showed the opposite pattern for modifier-biased trials, though the gap was smaller. Although Figure 3 appears to show differences in mouse trajectory for the two diagnostic groups, participants’ mouse movements were not significantly related to participants’ language proficiency or text exposure, but movements on control trials positively predicted their movements on experimental trials.

**TABLE 4:** Results of the mixed effects linear model for maximum deviation of mouse trajectories as influenced by consistency of choice with verb bias, choice of interpretation, measures of text exposure (title recognition task) and language proficiency (SPELT-3 standard score), and their interactions, as well as expected strength of verb bias and average maximum deviation on control trials.

**Factor**	**Variance**	***SD***	**β**	***SE***	***p***
**Random factors**					
Subject intercept	0.004	0.07			
Verb bias	0.07	0.26			
Consistency of choice	0.04	0.20			
**Fixed factors**					
(Intercept)			0.14	0.06	**0.01**
Verb bias (reference category = instrument)			0.11	0.09	0.20
Consistency of choice of interpretation (reference category = consistent)			0.14	0.10	0.15
SPELT-3 standard score, centered			–0.002	0.002	0.25
Title recognition task score, centered			0.004	0.003	0.17
Strength of verb bias			–0.002	0.001	0.18
Average maximum deviation on control trials			0.50	0.18	**0.01**
Verb bias × Consistency of choice			–0.29	0.15	**0.01**
Verb bias × SPELT			–0.003	0.005	0.51
Verb bias × Title recognition task			0.003	0.007	0.66
Consistency of choice × SPELT			0.0006	0.006	0.91
Consistency of choice × Title recognition task			0.008	0.008	0.33
Verb bias × Consistency of choice × SPELT			0.005	0.006	0.47
Verb bias × Consistency of choice × Title recognition task			–0.02	0.01	**0.09**

*Bold indicated significance (*p* = 0.05) or near significance (*p* < 0.1).*

## Study 1 Discussion

We found that children with and without DLD ages 7–9 were primarily influenced by global bias in their choice of pictures. However, children showed sensitivity to local verb bias information in their mouse movements. There was a greater deviation toward the unchosen picture when choosing against bias on instrument-biased trials than on modifier-biased trials.

It was not surprising that children chose instrument interpretations most often, given the global instrument bias. The global bias also likely contributed the different patterns of mouse trajectories between instrument- and modifier-biased verbs. In our previous study with college students using the same stimuli (Hall et al., 2019), participants also showed a preference for the instrument interpretation and similar mouse trajectory patterns, with stronger evidence of verb bias sensitivity for instrument-biased verbs. Audio and visual stimuli in our task may have influenced children’s choice differently from Snedeker and Trueswell’s (2004) study. That study showed that children were likely still learning to integrate visual and linguistic cues, with behavioral differences between adults and children when an additional visual cue was added. In fact, the eye tracking data from that study revealed that children’s eye movements were beginning to pattern more like adults,’ even though children’s choice of interpretation did not yet reflect this. The findings from Peter et al. (2015) also provide evidence for a longer developmental trajectory for verb bias/cue integration, with differences in verb bias effects among 3- and 6-year-olds and adults in a syntactic priming paradigm. It was surprising that for both choice of interpretation and mouse movements, we found no correlation with measures of language or text exposure. This suggests that perhaps other aspects of cognition, such as working memory or cognitive control (see for example, Just and Carpenter, 1992; Novick et al., 2005; Lewis et al., 2006; Martin, 2016), are driving the maturation of cue integration in sentence processing.

Being able to efficiently predict upcoming information in a linguistic signal may have a profound impact on overall comprehension. If children with DLD are slower and less efficient in making their predictions than typical peers, they risk missing crucial information for making timely connections during conversations. We have no evidence of group differences among children from this data set, but Hall et al. (2019) indicates that differences do exist in adulthood using the same task. This suggests that studies of adolescent language development may be important for fully understanding the functional differences observed in adult outcomes (Conti-Ramsden and Durkin, 2007; Carroll and Dockrell, 2010; Hesketh and Conti-Ramsden, 2013). The current study suggests that syntactic prediction during processing may not yet be adult-like at these ages given that typical children as well as children with DLD showed little evidence of verb bias in their choice of interpretation.

The main contribution of this study is to take a first step examining individual differences in verb bias sensitivity in children with and without DLD. In general, results suggest that children ages 7–9 with and without DLD do not consistently use verb bias information to resolve ambiguity, though they are sensitive to verb bias. Importantly for this paper, there is also sufficient variability in performance for consideration of how individual differences in statistical learning might contribute, despite (or perhaps because of) our finding that measures of language proficiency, and text exposure did not meaningfully predict verb bias sensitivity.

## Study 2 Introduction

In this study we adopt a “multiverse” approach (Steegen et al., 2016) to examine whether a particular statistical learning task predicts performance on the verb bias task described in Study 1. Data is drawn from the verb bias study reported in Hall et al. (2019) and the artificial grammar learning studies reported in Hall et al. (2018a,b), and Hall et al. (2017). We walk through some of the rationale for the different measurement choices first, and then present the findings.

## Study 2 Methods

### Participants

#### Adults

To ensure enough participants to adequately power a test of relationship, we added 31 additional TD adult participants to the dataset of the 33 adult participants from TD and DLD groups in the verb bias study reported in Hall et al. (2019) and the artificial grammar learning study reported in Hall et al. (2017). The additional 31 adult participants were recruited from the University of Iowa Elementary Psychology Research Exposure participant pool and were screened by self-report for being monolingual and having no history of language or cognitive impairment. All of the participants in this TD group met qualifying criteria on the tasks (at least 60% accuracy on the one-back task in the artificial grammar learning task, and at least 50% accuracy on comprehension trials in the verb bias task), and as such, data from all participants were included. Demographic information for all 64 adult participants are presented in Table 5, again with the participants from the original studies presented in the first two rows. For more information on how adult participants with DLD were identified, please see Hall et al. (2019).

**TABLE 5:** Participant demographic and testing means and standard deviations by diagnostic category (DLD, developmental language disorder; and TD, typically developing), after excluding participants as described in the screening measures in Hall et al. (2019) and Hall et al. (2017) for adult datasets in Study 2.

															**Author**	
							**KBIT-2**								**recognition**	
			**Age**		**Education**		**standard**		**Spelling**		**Token**		**PPVT-4**		**task**	
			**(years)**		**(years)**		**score**		**(out of 15)**		**(out of 44)**		**raw score**		**(−65 to 65)**	
									
	***n***	***n* male**	***M***	***SD***	**M**	***SD***	***M***	***SD***	***M***	***SD***	***M***	***SD***	***M***	***SD***	***M***	***SD***
DLD	17	8	20.7	1.1	13.9	1.1	99.1	10.5	4.2	2.6	35.2	5.5	199.4	11.2	16.4	12.7
Matched TD	16	8	21.0	1.9	14.1	1.9	108.9	12.3	11.7	2.5	40.1	3.1	206.8	6.8	18.9	7.0
Additional TD	31	14	20.8	1.5	*Not collected*		*Not collected*		*Not collected*		*Not collected*		202.5	10.3	24.8	15.1

*Scores on Kaufman Brief Intelligence Test, 2nd Edition, non-verbal subtest (KBIT-2) are standard scores with a normative mean of 100 and a standard deviation of 15. Scores on the spelling and token tasks are raw counts of items correct out of 15 and 44, respectively. Scores on the Peabody Pictures Vocabulary Test 4th Edition (PPVT-4) are raw scores. Scores on author recognition task (ART) are out of a range of −65 to 65, with 65 being the highest possible score.*

#### Children

Child participants are the same as those from Study 1, with the six children excluded in Study 1 also excluded here. Demographic information are presented in Table 1.

### Analysis

We used performance on the artificial grammar learning task as a continuous variable in a mixed effects linear model predicting performance on the verb bias task. There are many arbitrary ways to measure variables by which to look for a relationship between the tasks, which led us to adopt this “multiverse” approach (Steegen et al., 2016). Table 6 lists measures for each participant group, task, verb set, and dataset, with abbreviations used in the section “Results.” We included a random subject intercept in all models because the Akaike Information Criterion (AIC) indicated this was the best fit for the previous models we ran analyzing the verb bias data in both children and adults. We do not consider alternative random effects structures for the models for the sake of space, but we recognize that these also could impact findings. Code to run analyses in R version 3.5.1 (R Core Team, 2018) and sample data are available on github at https://github.com/jessica-hall/multiverse/.

**TABLE 6:** Choices of measures, definitions, and abbreviations for multiverse analysis.

**1. Participant datasets** Adult 1: Adult participants with DLD and TD Adult 2: Adult TD participants only Child 1: Child participants with DLD and TD Child 2: Child TD participants only **2. Verb bias measures** VB1: Mean consistency of choice of interpretation on 0–1 scale; 0 = inconsistent, 1 = consistent; logistic regression model VB2: Maximum deviation (MD), interaction with consistency as a categorical variable; all trials; linear regression VB3: MD, interaction with consistency; instrument-biased trials only; linear regression VB4: MD instrument-biased trials with choice of interpretation = modifier; linear regression **3. Verb sets** S1: Full set of verbs S2: Full set of verbs and strength variable S3: Strongly biased verbs only S4: Weakly biased verbs only **4. Artificial grammar learning measures, difference in mean standardized rating of each item type** AGL1: Novel minus ungrammatical, entire test AGL2: Novel minus ungrammatical, first half of test

#### Participants to Include

For all models, we tested both adults and children (Adult 1 and Child 1 in Table 6) and we also tested a subgroup dataset of TD participants only (Adult 2 and Child 2). We did this because we found group differences in verb bias sensitivity in adults (Hall et al., 2019), and thus the participants with DLD may have relied on other information to perform the verb bias task and therefore would not show a relationship between performance on both tasks.

#### Dependent Variable: Verb Bias Measure

We had two types of measures for this task, and thus we consider two types of dependent variables in our models: choice of interpretation and mouse trajectories. The measure for choice of interpretation is consistency with verb bias, dummy coded as “1” for consistent and “0” for inconsistent, in a logistic regression analysis (VB1 in Table 6). Thus, the hypothesis tested in these models is whether good learning in the artificial grammar learning task predicts *responses consistent with verb bias* in the verb bias task. The rationale here is that because the statistical learning task requires explicit evaluation of items, perhaps it will show more relation to the more explicit decision of which interpretation participants choose. For the mouse trajectories, we consider consistency of choice of interpretation as an independent variable that interacts with the statistical learning measure, with the maximum deviation of the mouse trajectory value as the dependent variable (VB2). An interaction between these variables would indicate a relationship between distributional learning in an artificial setting and distributional learning in the real world. The hypothesis tested in these models is whether good learning in the artificial grammar learning task predicts *more attraction to the unselected response* when choosing an interpretation inconsistent with verb bias. The rationale for using this dependent variable is that the mouse trajectory measure can capture a wider spectrum of differences in sentence processing than simply which picture participants chose and therefore will be a more sensitive measure of individual differences.

Next, there are several possibilities to consider for which trials to include. One is the full dataset. A second is a dataset restricted to instrument-biased verbs only because in both child and adult studies, participants showed more sensitivity to the bias of instrument-biased verbs (VB3). A third alternative is to restrict the dataset further to only instrument-biased trials in which participants chose a modifier interpretation, because choosing modifier on instrument-biased trials is the instance in which we expect to see the greatest evidence of verb bias sensitivity (greater maximum deviation values; VB4). In the most restricted models, then, there is no covariate measure of consistency of choice of interpretation because we are only considering one choice.

Finally, because we found a relationship with the expected strength of verb bias according to the norming data provided by Snedeker and Trueswell (2004), we test each of these alternatives using the full set of verbs (S1 in Table 6), using the full set of verbs with a strength interaction term (S2), and using a restricted set of only the strongly biased verbs for the adults (S3) or only the weakly biased verbs for the children (S4). Table 2 provides strength of verb bias ratings for each verb. We switch from strongly biased verbs (S3) for adults to weakly biased verbs (S4) for children because examination of data from Study 1 indicated that children showed stronger verb bias effects for weakly biased verbs, in contrast to the pattern that adults showed in Hall et al. (2019). The rationale for including this measure is that it allows us to capture some of the variability that may be associated with the linguistic element of the stimuli rather than the visual elements and therefore represent a clearer picture of the role of verb bias in sentence processing.

#### Independent Variable: Statistical Learning Measure

Because performance on the statistical learning task has been described with these same participant datasets, we provide only a brief description of what is to be learned in the task and review prior results briefly.

In this task, participants listened to an artificial language that contained “gaps” of information. In the language, words of the same “category” had similar, but not perfectly overlapping, distributions. This task provides a good approximation of the type of learning required for verb bias. In the case of verb bias, one deduces subcategorizations from hearing sets of verbs appear in similar but not perfectly overlapping distributions. Similarly, in this artificial grammar learning task, one must attend to the item and the way in which it distributes into syntactical contexts and how those are interpreted in order to deduce categories and succeed at learning the grammar. At test, novel grammatical items contained combinations that were not heard during exposure but that were grammatically possible according to the shared distributional features, as well as ungrammatical items that contained unheard grammatically impossible distributional features. The key test of learning in the task is the difference in participants’ ratings of novel grammatical and ungrammatical test items. Participants rated items on a visual analog scale that we translated to values from 0 to 100, with 0 being ungrammatical and 100 being grammatical. We used *z* values to create a standardized measure for each participant because there was individual variation in how the scale was used. The simplest measure of distributional learning in the artificial grammar learning task is to take the difference in average ratings for novel grammatical and ungrammatical test items (AGL1 in Table 6). A large positive difference in ratings between novel and ungrammatical items would indicate learning of the grammar. As reported in Hall et al. (2018b), we found that on average, child and adult participants with and without DLD rated novel items higher than ungrammatical items, with no differences between diagnostic or age groups.

However, because we found an effect of testing order (items tested earlier received higher ratings than items at the end of the test), we also considered a separate measure of the difference in ratings for novel, and ungrammatical items from the first half of the test only (AGL2 in Table 6). Adding order to our model in our previous study distinguished both diagnostic groups and age groups. As we discuss in Hall et al. (2018b), the order effect may indicate sensitivity to a changing distribution as ungrammatical items are heard during the test phase. It is possible that in averaging ratings for novel items *throughout* the test, we would have means for each participant near zero because of positive ratings early on and negative ratings later. We would not be able to distinguish learners who showed strong order effects and learners who always rated grammatical novel items near the midpoint of the scale (zero). This is an important distinction to make because we would consider the former to be good at distributional learning and the latter not as good. Therefore, we include two measures of statistical learning, one from the entire test and the other with items from first half only. General performance was such that 15 of 16 children with DLD, 20 of 24 TD children, 17 of 17 adults with DLD, and 14 of 17 TD adults had a positive difference in mean ratings in items over the entire test, indicating learning. The amount of difference ranged from −0.19 to 1.5 for the children and −0.37 to 1.61 for the adults. These numbers changed minimally within each group when considering the first half of the test only.

#### Summary of Multiverse Methodology

To summarize, we consider a total of 48 models for each age group, adults and children: for the choice of interpretation as depedendent variable model, we have 2 participant subsets × 3 strength of verb bias measures × 2 artificial grammar learning measures. For the mouse trajectory as dependent variable model, we have 2 participant subsets × 3 strength of verb bias measures × 3 measures of mouse trajectory × 2 artificial grammar learning measures.

## Study 2 Results

### Children

**FIGURE 4:**
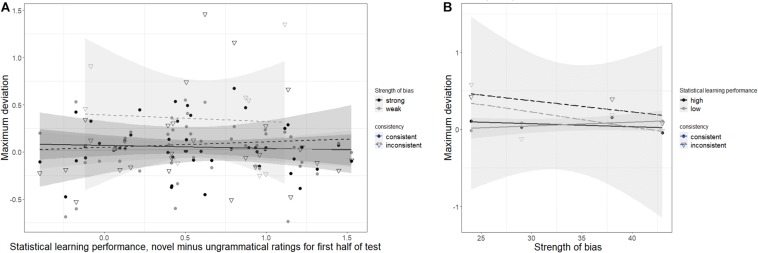
The amount that children showed sensitivity to verb bias, in that choosing inconsistently with bias (dashed lines) resulted in more curved trajectories (increased maximum deviation) relative to choosing consistently with bias (bold lines), particularly for weakly biased verbs, was predicted by performance on the statistical learning task, as measured by the difference in standardized ratings between novel and ungrammatical items on the artificial grammar learning task. **(A)** Statistical learning performance plotted continuously on the *x*-axis with strength of bias as a categorical variable (strong verbs are those rated above 29 in Table 2). **(B)** Strength of bias plotted on the *x*-axis with statistical learning performance as a categorical variable (high learners are above the median difference and low learners are below). Shading represents the standard error of the model.

We list beta estimates, standard errors, and *p* values for all of the critical effects (as explained above) as well as the number of participants and number of observations for each model in Table 7. None of the 48 models with child participants returned any significant result for the critical effects. Two models were marginally significant, *p* < 0.10. One possibility is that there is, in fact, no relationship between this AGL task and the verb bias measures. Another is that selection of the proper comparisons is critical for observing the anticipated result. The two marginally significant models were models that had mouse trajectories (as measured by maximum deviation) as the dependent variable and had instrument-biased verbs only (VB3), and included a variable for strength of verb bias (S2). The statistical learning measure was for the first half of the test only (AGL2). The results were similar for each of the two datasets run, one with all participants (Child 1, *p* = 0.09), and the other with only TD participants (Child 2, *p* = 0.07) and are reported in Supplementary Materials. For these models, children with higher statistical learning performance showed more curved trajectories when their choice was inconsistent than when their choice was consistent. This effect was true for weakly biased verbs. Strongly biased verbs actually showed the opposite pattern, with much greater curvature overall for consistent trials compared with inconsistent trials. Figure 4 presents these interactions in two plots to fully illustrate these effects. Figure 4A shows the continuous effect of statistical learning performance and Figure 4B demonstrates the continuous effect of verb bias strength. As can be seen most clearly in Panel B children with high statistical learning performance (in black) showed a stronger verb bias effect for weakly biased verbs (a larger gap between the dashed and bold lines) than strongly biased verbs, and children with low statistical learning performance (in gray) did not demonstrate a verb bias effect for strongly biased verbs. Note that in Figure 4A, for both very low and very high statistical learning performance, children never chose inconsistently with bias for weakly biased verbs, most easily seen by the narrower (but taller) shaded area around the dashed gray line. This restriction of range for certain item types may explain the marginal significance for these models.

**TABLE 7:** Beta estimates (β), standard errors (SE), and *p* values for the critical variable of each model, and number of participants (*n*) and observations (*n* obsv) for each model run for child datasets.

**Verb bias task measures and verb sets**	**Participant datasets and artificial grammar learning task measures**															
	
	**Child 1**								**Child 2**							
						
	**AGL 1**			**AGL 2**					**AGL 1**			**AGL 2**				
								
	**β**	**SE**	***p***	**β**	**SE**	***p***	***n***	***n* obsv**	**β**	**SE**	***p***	**β**	**SE**	***p***	***n***	***n* obsv**
**VB1**																
S1	–0.17	0.24	0.48	–0.04	0.16	0.82	49	678	–0.13	0.28	0.63	0.09	0.18	0.63	33	470
S2	0.01	0.02	0.53	–0.01	0.01	0.36	49	678	0.02	0.02	0.42	–0.009	0.01	0.51	33	470
S4	–0.28	0.34	0.40	0.11	0.22	0.62	49	342	–0.09	0.40	0.83	0.35	0.25	0.17	33	237
**VB2**																
S1	0.06	0.11	0.56	0.07	0.07	0.33	49	678	–0.03	0.12	0.83	–0.02	0.08	0.79	33	470
S2	0.002	0.007	0.78	0.006	0.01	0.25	49	678	0.005	0.01	0.56	0.007	0.005	0.16	33	470
S4	0.13	0.15	0.40	0.05	0.1	0.64	49	342	–0.11	0.16	0.49	–0.14	0.1	0.18	33	237
**VB3**																
S1	0.08	0.20	0.68	–0.01	0.14	0.97	49	337	0.08	0.23	0.72	–0.13	0.15	0.39	33	234
S2	0.009	0.03	0.72	0.03	0.02	**0.09**	49	337	0.02	0.03	0.57	0.04	0.02	**0.07**	33	234
S4	0.01	0.36	0.97	–0.29	0.29	0.33	48	126	–0.04	0.32	0.89	–0.44	0.27	0.10	33	89
**VB4**																
S1	0.19	0.23	0.42	0.02	0.16	0.92	32	50	0.25	0.28	0.37	–0.15	0.17	0.39	21	34
S2	0.003	0.03	0.92	0.02	0.02	0.21	32	50	0.009	0.03	0.79	0.03	0.02	0.21	21	34
S4	0.20	0.44	0.66	–0.25	0.36	0.50	10	14	0.19	0.55	0.74	–0.41	0.44	0.37	8	11

*Definitions of abbreviations can be found in Table 6. Bold indicated significance (*p* = 0.05) or near significance (*p* < 0.1).*

### Adults

**FIGURE 5:**
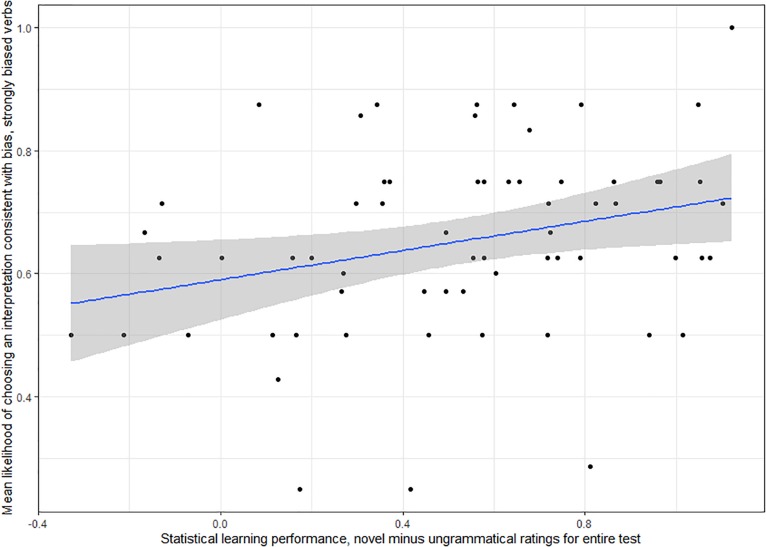
For adult participants, sensitivity to verb bias, as measured by the likelihood of choosing an interpretation consistent with bias on trials with strongly biased verbs, was predicted by statistical learning ability, as measured by the difference in standardized ratings between novel and ungrammatical test items on the artificial grammar learning task. Shading represents the standard error of the model.

Table 8 displays beta estimates, standard errors, and *p* values for all of the critical effects as well as the number of participants and number of observations for each model. Of the 48 models with adult participants, only one returned significant results for the critical effects, *p* = 0.054. This was the model with the difference in ratings for novel and ungrammatical items for the whole testing period (AGL1) on the artificial grammar learning task predicting the likelihood of a response consistent with verb bias (VB1), with only strongly biased verbs included (S3) and the dataset with all participants (Adult1 dataset). We report results for this model in Table 9 and Figure 5 provides an illustration.

**TABLE 8:** Beta estimates (β), standard errors (SE), and *p* values for the critical variable of each model, and number of participants (*n*) and observations (*n* obsv) for each model run for adult datasets.

**Verb bias task measures and verb sets**	**Participant datasets and artificial grammar learning task measures**															
	
	**Adult 1**								**Adult 2**							
						
	**AGL1**			**AGL2**					**AGL1**			**AGL2**				
								
	**β**	**SE**	***p***	**β**	**SE**	***p***	***n***	***n* obsv**	**β**	**SE**	***p***	**β**	**SE**	***p***	***n***	***n* obsv**
**VB1**																
S1	0.04	0.07	0.59	–0.001	0.07	0.98	64	930	0.03	0.08	0.71	0.01	0.08	0.89	47	688
S2	0.007	0.006	0.19	0.001	0.006	0.82	64	930	0.03	0.007	**0.058**	0.008	0.006	0.18	47	688
S3	0.19	0.10	**0.054**	0.09	0.10	0.35	64	469	0.20	0.11	**0.07**	0.13	0.11	0.23	47	348
**VB2**																
S1	–0.002	0.03	0.94	0.008	0.03	0.76	64	930	0.03	0.03	0.35	0.03	0.03	0.28	47	688
S2	0.001	0.002	0.50	0.001	0.002	0.74	64	930	0.001	0.002	0.69	0.001	0.002	0.59	47	688
S3	0.02	0.04	0.60	0.02	0.04	0.61	64	469	0.06	0.04	0.20	0.05	0.04	0.28	47	348
**VB3**																
S1	0.004	0.05	0.92	–0.01	0.05	0.81	64	472	0.01	0.05	0.85	–0.04	0.05	0.47	47	349
S2	–0.003	0.006	0.59	–0.002	0.006	0.7	64	472	0.0003	0.007	0.97	0.002	0.007	0.82	47	349
S3	–0.007	0.06	0.90	–0.02	0.06	0.72	64	291	0.004	0.07	0.96	–0.05	0.07	0.44	47	216
**VB4**																
S1	–0.02	0.06	0.73	–0.001	0.06	0.98	41	75	–0.02	0.07	0.75	0.03	0.07	0.70	29	49
S2	–0.001	0.008	0.86	–0.001	0.007	0.92	41	75	–0.003	0.009	0.75	–0.001	0.008	0.93	29	49
S3	–0.04	0.06	0.53	–0.01	0.07	0.85	36	53	–0.04	0.07	0.61	0.02	0.08	0.79	25	36

*Definitions of abbreviations can be found in Table 6. Bold indicated significance (*p* = 0.05) or near significance (*p* < 0.1).*

**TABLE 9:** Results for mixed effects logistic regression of factors predicting the probability of a choice consistent with bias in the verb bias task by adult participants, with a dataset that included only strongly biased verbs.

**Factor**	**Variance**	***SD***	***B***	**SE**	***p***
**Random factors**					
Subject	0.00	0.00			
**Fixed factors**					
(Intercept)			0.65	0.10	**<0.0001**
Artificial grammar learning performance (novel – ungrammatical, entire test)			0.19	0.10	**0.054**

*Bold indicated significance (*p* = 0.05) or near significance (*p* < 0.1).*

A similar model for the dataset with only TD participants (Adult 2) was marginally significant, *p* = *0.07*. Finally, one additional model with only TD participants which also included these variables and an interaction with strength rather than a subset of strongly biased verbs (S2) was borderline at *p* = 0.058. Results for these models are reported in Supplementary Materials. All models show a trend for participants with higher statistical learning scores more likely to choose interpretations consistent with verb bias on the verb bias task than participants with lower statistical learning scores.

## Study 2 Discussion

Our previous studies (Hall et al., 2017, 2018a,b) demonstrated that children and adults with DLD are capable of learning from distributional dependencies in an artificial grammar learning task similarly to their TD peers. However, there was considerable spread in performance by all groups. We predicted that how well individuals learned distributional dependencies in the artificial language task would have bearings on how well they use distributional information to resolve ambiguous sentences in real language. We found some evidence that individual differences in statistical learning predicted performance on the verb bias task in adult participants but not in children, but the findings are not robust. This was not especially surprising given that adults with DLD showed differences from TD peers on the verb bias task (see Hall et al., 2019), but Study 1 did not demonstrate that individual differences in language proficiency or text exposure predicted performance by child participants. The relationship was found only for predicting consistency of choice of interpretation and not for mouse trajectories in the adult participants. Indeed, children did not appear to be using verb bias when choosing an interpretation, and thus this may be why we did not see this relationship for them. That the relationship in adults seemed to be driven by the TD participants provides further evidence that adults with DLD are not using distributional information in the same way as TD peers when disambiguating sentences in the verb bias task. It is possible that some of the adults with DLD, like the child participants, were not using verb bias to disambiguate the sentences in the sentence processing task.

At the suggestion of a reviewer, we examined the internal consistency coefficient (ICC) for our primary independent and dependent measures in the significant model. As might be expected from previous results that showed large standard deviations for ratings in the statistical learning task with typical children (Hall et al., 2018a), we obtained very low measures of internal consistency for the artificial grammar learning task measures. Because the measure for of statistical learning depended on ratings for novel items, it was likely impacted by the great amount of noise, even in the adult data. We also obtained low measures of reliability for the verb bias task measures. The poor values on these measures of reliability conducted *post hoc* suggest that the tasks are not well suited for measuring individual differences, at least for the number of items on the tasks in the present study. Low reliability may explain why most of the models in our multiverse analysis were not significant.

### Individual Differences

Given the number of comparisons run, it is reasonable to question whether the results obtained were simply spurious effects. Indeed, we would feel more confident if there was a more consistent result regardless of how the measures were selected. The value of the multiverse analysis is to demonstrate the robustness of the findings; because the majority of models were not significant, the significant findings from this study are not robust. Nonetheless, we believe it to be valuable to reflect on whether there is a rational explanation for why three comparisons reached or were near significance for the adults and the remainder were not.

First, the two verb bias task measures differed on the degree of explicitness. The choice of interpretation was likely a better match for the explicit grammaticality test in the artificial grammar learning task than the implicit mouse trajectory measure. A more implicit measure of learning in the artificial grammar learning task (such as that in Lammertink et al., 2019, and López-Barroso et al., 2016) may have better predicted mouse trajectories on the verb bias task. Other studies with implicit measures have found positive relationships with children; for example, Kidd (2012) showed that 4- and 5-year-olds’ performance on serial reaction time task corresponded with their ability to remember a primed sentence structure. It is beyond the scope of this paper to delineate whether implicit and explicit processes are discrete processes, but we suggest this as an area worthy of further investigation.

Second, the choice of interpretation may have been more impervious to factors like motivation, alertness, or attentiveness that may have added to the variability in mouse trajectories. The choice of interpretation may not have changed much because it may not require much effort to understand the sentences, but the speed and dexterity of mouse movements could change dramatically over time as participants become more fatigued or bored with the experiment, or, in the case of children, more adjusted to using the mouse (some had never seen one before). We might also attribute mouse trajectories as reflecting the ability to use distributional information to do speeded language processing. In the artificial grammar learning task, participants are not given a time limit to make their grammaticality decisions. In addition, the cognitive load is fairly low: participants listen to a three-word “sentence” and hold it in memory as they compare it to stored mental representations for items heard five to fifteen minutes earlier. They are not told to remember any specific items or even asked if the sentence is the same as ones heard previously, easing the task to some extent. The verb bias task, on the other hand, requires that participants listen to a somewhat complex sentence while looking at visually complex stimuli and then move as quickly as possible to one of two pictured interpretations. Differences in mouse movements therefore may have been more affected by differences in executive function or speed of processing. This could have had the effect of stabilizing the within-participant variability, and in fact the adults had greater within-participant variability on the choice of interpretation measures than the mouse trajectory measures.

Also of note is that the internal consistency of the measures is low likely in part because of the low number of items. We obtained a large number of negative ICC values across all measures, indicating great within-subject variability for both statistical learning and sentence processing measures. For measures with positive values, the number of items was often quite low (restricting to weakly biased verbs halves the dataset, and further restricting it to instrument-biased verbs halves it again; see Tables 7, 8 for number of observations within each model). With more items, tasks might have been more reliable, and therefore more suited to showing a strong relationship. Although the study of the psychometric properties of statistical learning tasks is in its infancy, reliability-related issues may contribute to the difficulty in identifying experimental links between statistical tasks and measures of online language processing, especially with child participants (Arnon, 2019). It is possible better reliability may be obtained through larger numbers of test items, but adding more items will impact the age of potential child participants capable of completing tasks. There is also the problem of increasing participants’ exposure to ungrammatical combinations with more test items in an artificial grammar learning task, which could influence results and how results are interpreted.

Attention to the psychometric properties of statistical learning and online comprehension tasks would strengthen the inferences possible in this field. As noted by Arnon (2019), increasing the type of items tested (Siegelman et al., 2017) and using online methodologies (Siegelman et al., 2018b) are two ways to improve the reliability of statistical learning tasks without fatiguing child participants. We hope to take steps in future research that will allow us to improve reliability in these types of measures when used with young children.

### Developmental Differences

We found clear diagnostic group differences by adults in the verb task (Hall et al., 2019) but not in the artificial grammar learning task (Hall et al., 2017). This may have been due to greater task demands in the verb bias task related to the complexity of real language and the variation in individuals’ experience with language. Although the language that people with DLD hear may not differ substantially from what people with TD hear (Leonard, 2014; though Karmiloff-Smith, 2009, has questioned whether impairments or deficits are compounded upon by how others interact with individuals with developmental disorders), the accumulated experience of DLD may result in a linguistic experience that is not as rich or deep as peers. For example, difficulty learning language could result in weaker semantic and syntactic representations (see Sheng and McGregor, 2010; Alt et al., 2013; Haebig et al., 2017), which then limit the expressiveness of the individual’s own speech, as well as the efficiency and the precision with which the individual understands others’ speech. This may have a cascading effect, such that weak representations in childhood limit how later information is stored – even if the child is exposed to the same information – or may lead to the child seeking out different types of interactions as they age, leading to actually different experiences and interactions in adolescence. In the artificial grammar learning task, on the other hand, the language is tightly controlled. There are no competing experiences, the learning is not under time pressure, the exposure period is small, and all participants have identical exposures. There is little variation; in short, it is a toy language, and the controlled nature of the language likely made it an easier task than the verb bias task for all participants.

Snedeker and Trueswell (2004) demonstrated that verb bias cues outweighed referential cues for 5-year-old children, which differentiated them from the adult participants in their study of verb bias. Although children made choices based squarely on verb bias, their eye movements indicated emerging consideration for referential cues (the number of animals present influenced how often they looked at objects indicating a modifier interpretation). It is possible that at ages 7–9, children are now learning to integrate and weight different cues during sentence processing and not relying so squarely on verb bias. However, the process is not complete, and so we still see differences between them and adults. That we see the opposite pattern of Snedeker and Trueswell (2004) should not be surprising because we did not have referential cues here. And so, although we know that children are capable of learning from distributional information similarly to adults (Hall et al., 2018b) and that they are sensitive to the different distributional properties of verbs in the task we used, we can infer that children show different patterns of interpretation than adults in both their mouse movements and overt choices because they are in the process of learning to use other cues to interpret sentences.

Regarding development and verb bias, it is possible that our tasks were not as similar as we hoped, in that although they both involved distributional information, our statistical learning task involved tracking adjacent dependencies (which words occurred next to each other) whereas verb bias, in this case, was a non-adjacent dependency (a noun appeared between the verb and the ambiguous *with the x* phrase). It is possible that working memory or other cognitive limitations could have impacted children’s performance on the verb bias task differently from the statistical learning task (e.g., Thiessen et al., 2013), and that these differences may have been better captured in a task that tracked learning of non-adjacent distributional information. This is an example of how a specific type of statistical learning may be more relevant for an emerging skill at this point in development than another.

It is important to continue to study distributional learning using both real and artificial language stimuli to better understand the mechanisms involved and how they might facilitate grammar acquisition and use in typical populations and in populations with DLD. Furthermore, both cross-sectional and longitudinal studies should assess performance in the intervening periods between early childhood and adulthood to better elucidate the developmental trajectories of both typical and atypical populations (McMurray et al., 2010, 2018; Rigler et al., 2015).

## General Discussion

In the present work, we examined the roles of grammatical proficiency, text exposure, and statistical learning for explaining individual differences in sentence processing by children and adults with and without DLD. Our general purpose was to better understand the relationship between statistical learning and language learning and processing. In Study 1, we found that children with DLD and their TD peers showed some sensitivity to verb bias in an implicit mouse tracking measure even when their explicit behavior did not reflect this sensitivity. Instability in the formation of verb biases as part of typical development may have contributed to the pattern of findings for children. In our second study, we found that our measure of statistical learning predicted how adults interpreted ambiguous sentences using verb bias in only one of 48 possible models. It is possible that we saw stronger evidence for a relationship for TD adults than adults with DLD because adults with DLD use verb bias information differently or do not have the same access to the information during sentence processing as TD peers. However, findings for a relationship were not robust and reliability for all measures was quite low. Together, these results suggest that language processing is influenced by the statistics in the language environment and one’s ability to attend to them and use them, but we need more reliable tasks to better detect and understand this relationship. The performance by children in comparison to adults on the verb bias task, taken in combination with the findings on statistical learning, suggests that there may be differences between initial learning of statistical information in linguistic environments and using that information efficiently during complex language processing combined with other cues.

While we acknowledge some uncertainty about which aspects of the verb bias stimuli may have affected how children interpreted sentences, results from Study 1 provide novel insights. We now know that, although children with and without DLD may be sensitive to verb bias information, the global instrument bias and integration of visual and linguistic cues also affects how verb bias information is deployed during processing, and likely has a long developmental timeline. Unique contributions of Study 2 are that TD adults may rely on the same mechanisms to learn from distributional information and to predict distributional information while processing language. These results provide further evidence that statistical learning may contribute to variation in how individuals process and interpret language, at least in adulthood. These studies allow more nuanced discussion of the mechanisms responsible for efficient sentence processing and the developmental timescales of these mechanisms.

Results from these studies provide further evidence that verb bias continues to develop beyond school age, and that differences observed in adults with and without DLD suggest that that verb bias is an area of weakness in DLD, albeit one that may appear somewhat hidden until later in development when verb biases and cue integration are more fully formed in the TD population. Given the impact that verb bias can have on comprehension and communication, it is an area worthy of further study.

Results from Study 2 illustrate a need for more transparent methods for reporting results from studies of complex mechanisms, such as those purported to support multifaceted skills like language. Because *post hoc* explanations of data are tempting and often seem rational given trends in the literature, we recommend best practice be either using highly transparent methodology such as multiverse analyses or preregistering both the tasks chosen and the final measures along with potential explanations and predicted outcomes to reduce the temptation to report only the most exciting findings. We also encourage researchers to examine tasks’ psychometric properties and report measures of reliability in studies of statistical learning and language processing. For progress in research into the cognitive science of language, a commitment to open science is necessary to ensure that results can be verified and replicated. Without extensive reporting of how and why variables were chosen and measured, our work will always be exploratory.

## Data Availability Statement

Code to run analyses in R version 3.5.1 (R Core Team, 2018) and sample data are available on github at https://github.com/jessica-hall/multiverse/. Full datasets generated for this study are available on request to the corresponding author.

## Ethics Statement

The studies involving human participants were reviewed and approved by the University of Iowa Institutional Review Board. Written informed consent to participate in this study was provided by the participants’ legal guardian/next of kin.

## Author Contributions

JH was the lead author of this manuscript, contributed the majority of the writing during her postdoc at the University of Arizona, conducted the experiments, and analyzed data as part of her dissertation requirement while a student at the University of Iowa. AO and TF provided substantial insight and ideas during the development of stimuli and data analysis. AO contributed to the writing and editing of drafts of these datasets, and provided the insight into developmental language disorder at different ages as well as access to populations seen in her labs.

## Conflict of Interest

The authors declare that the research was conducted in the absence of any commercial or financial relationships that could be construed as a potential conflict of interest. The reviewer GS-C declared a shared affiliation, with no collaboration, with one of the authors, TF, to the handling Editor at the time of review.
