# Supplementary material for: Individual Differences in Verb Bias Sensitivity in Children and Adults With Developmental Language Disorder
Source: Front Hum Neurosci. 2019 Nov 19;13:402. doi: 10.3389/fnhum.2019.00402 (PMC6877742; doi:10.3389/fnhum.2019.00402)
Supplement: Supplementary file 1 [file Table_1.pdf]

Supplementary material

Table S1. Results for mixed effects model for factors predicting the maximum deviation of the mouse trajectory in the verb bias task by all child participants, with a dataset that included a continuous variable of bias strength, a categorical variable for consistency of choice, and performance on the first half of the statistical learning task.

Factor	Variance	SD	*B*	*SE*	*p*
Random Factors					
Subject	.01	.09			
Fixed Factors					
(Intercept)			-.07	.12	.57
Artificial grammar learning performance (novel – ungrammatical, first half of test)			.33	.24	.17
Strength, measured continuously			.0002	.003	.96
Consistency of choice with verb bias (consistent=reference variable)			.87	.32	.01
Artificial grammar learning x strength			-0.01	.007	.14
Artificial grammar learning x consistency			-1.14	.72	.11
Consistency x strength			-0.02	.009	.02
Artificial grammar learning x strength x consistency			.03	.02	**.09**

Table S2. Results for mixed effects model for factors predicting the maximum deviation of the mouse trajectory in the verb bias task by typically developing child participants, with a dataset that included a continuous variable of bias strength, a categorical variable for consistency of choice, and performance on the first half of the statistical learning task.

Factor	Variance	SD	*B*	*SE*	*p*
Random Factors					
Subject	.01	.09			
Fixed Factors					
(Intercept)			-.009	.13	.94
Artificial grammar learning performance (novel – ungrammatical, first half of test)			.34	.25	.18
Strength, measured continuously			.002	.004	.60
Consistency of choice with verb bias (consistent=reference variable)			.69	.35	.051
Artificial grammar learning x strength			-0.01	.01	.18
Artificial grammar learning x consistency			-1.39	.74	.06
Consistency x strength			-0.02	.01	.08
Artificial grammar learning x strength x consistency			.04	.02	**.07**

Table S3. Results for mixed effects logistic regression of factors predicting the probability of a choice consistent with bias in the verb bias task by typically developing adult participants, with a dataset that included only strongly-biased verbs.

Factor	Variance	SD	*B*	*SE*	*p*
Random Factors					
Subject	.00	.00			
Fixed Factors					
(Intercept)			.74	.12	<.0001
Artificial grammar learning performance (novel – ungrammatical, entire test)			.20	.11	**.07**

Table S4. Results for mixed effects logistic regression of factors predicting the probability of a choice consistent with bias in the verb bias task by typically developing adult participants, with a dataset that included a continuous variable of bias strength.

Factor	Variance	SD	*B*	*SE*	*p*
Random Factors					
Subject	.00	.00			
Fixed Factors					
(Intercept)			-.40	.24	.09
Artificial grammar learning performance (novel – ungrammatical, entire test)			-.35	.22	.1
Strength, measured continuously			.03	.007	<.0001
Artificial grammar learning x strength			.01	.006	**.058**
